# The mutation rate of *rpoB* gene showed an upward trend with the increase of MIRU10, MIRU39 and QUB4156 repetitive number

**DOI:** 10.1186/s12864-023-09120-y

**Published:** 2023-01-16

**Authors:** Fan Su, Lei Cao, Xia Ren, Jian Hu, Grace Tavengana, Huan Wu, Yumei Zhou, Yuhan Fu, Mingfei Jiang, Yufeng Wen

**Affiliations:** 1 School of Public Health, Wannan Medical College, Wuhu, Anhui Province China; 2 School of Laboratory Medicine, Wannan Medical College, Wuhu, Anhui Province China; 3 School of Clinical Medicine, Wannan Medical College, Wuhu, Anhui Province China

**Keywords:** *RpoB* gene, Mutation, MIRU10, QUB4156, MIRU39, Repetitive number

## Abstract

**Background:**

Mycobacterial interspersed repetitive unit-variable number tandem repeat (MIRU-VNTR) is a frequently used typing method for identifying the Beijing genotype of *Mycobacterium tuberculosis* (*Mtb*), which is easily transformed into rifampicin (RIF) resistance. The RIF resistance of *Mtb* is considered to be highly related with the mutation of *rpoB* gene. Therefore, this study aimed to analyze the relationship between the repetitive number of MIRU loci and the mutation of *rpoB* gene.

**Methods:**

An open-source whole-genome sequencing data of *Mtb* was used to detect the mutation of *rpoB* gene and the repetitive number of MIRU loci by bioinformatics methods. Cochran-Armitage analysis was performed to analyze the trend of the *rpoB* gene mutation rate and the repetitive number of MIRU loci.

**Results:**

Among 357 rifampicin-resistant tuberculosis (RR-TB), 304 strains with mutated *rpoB* genes were detected, and 6 of 67 rifampicin susceptible strains were detected mutations. The *rpoB* gene mutational rate showed an upward trend with the increase of MIRU10, MIRU39, QUB4156 and MIRU16 repetitive number, but only the repetitive number of MIRU10, MRIU39 and QUB4156 were risk factors for *rpoB* gene mutation. The Hunter-Gaston discriminatory index (HGDI) of MIRU10 (0.65) and QUB4156 (0.62) was high in the overall sample, while MIRU39 (0.39) and MIRU16 (0.43) showed a moderate discriminatory Power.

**Conclusion:**

The mutation rate of *rpoB* gene increases with the addition of repetitive numbers of MIRU10, QUB4156 and MIRU39 loci.

## Introduction

In recent years, the global tuberculosis (TB) epidemic continues to be serious. Drug-resistant tuberculosis, especially those resistant to rifampicin (RR-TB), has become one of the major obstacles to achieve the goal of TB elimination [1]. According to the Global Tuberculosis Report 2020 of World Health Organization (WHO), there were an estimated 465,000 (range, 400,000–535,000) incident cases of RR-TB, and China accounts for 14% of them [2].

More than 95% of rifampicin resistance is associated with mutations in the *rpoB* gene of *Mycobacterium tuberculosis* (*Mtb*), with 97% of mutations occurring within the 81 bp rifampicin-resistant determining region (RRDR) of this gene [3]. Besides, it has been proved that sequence mutation out of RRDR may be involved in the formation of rifampicin cross resistance [4]. In China, Beijing genotype tuberculosis occupies a dominant position of *Mtb*, Uddin MKM et al. [5] had proved the mutation of *rpoB* gene was a risk factor of rifampicin resistance for Beijing genotype TB.

In recent years, mycobacterial interspersed repetitive units-variable number tandem repeats (MIRU-VNTR) had been widely used in the typing of TB. Combined with Spoligotyping, MIRU-VNTR typing can distinguish Beijing family genotype with other genotype strains by cluster analysis [6]. Besides, different MIRU loci showed different discriminatory power for Beijing and non-Beijing genotype strains and significant differences were found in mutation of the *rpoB* gene between two genotype [7, 8]. On this basis, we hypothesized that there may be a correlation between the mutation of *rpoB* gene and the repetitive number of MIRU loci.

With the popularization of whole-genome sequencing technology, the mutation of known drug resistance genes and MIRU-VNTR information can be obtained based on the analysis of *Mtb* Illumina, Pacific Biosciences or Oxford Nanopore sequencing data [9, 10]. Therefore, we conducted this study to explore the relationship of *rpoB* gene mutation and MIRU loci with sequencing data.

## Methods

### Data source

Sample information was acquired from one study of the Chinese Center for Disease Control and Prevention (Chinese CDC) [11], including the phenotypic drug resistance of each strain, type of patient from which the strain originated, etc. Whole-genome sequencing raw data were deposited at NCBI Sequence Read Archive (SRP134826) and Genome Sequence Archive (CRA000786) (https://ngdc.cncb.ac.cn/search/?dbId=gsa&q = CRA000786)

### RpoB gene mutation determination

In the first step, the sequencing data was submitted to remove linker and low-quality base treatment (filtering the bases with Phred < 20) using Fastp (https://github.com/OpenGene/fastp) software. Secondly, BWA (http://bio-bwa.sourceforge.net/bwa.shtml) software was used to compare the above sequence data with the genome template sequence of *Mycobacterium tuberculosis* standard strain (H37Rv) (obtained from the gene sequence database GenBank access: NC 000962.3 maintained by the National Institutes of Health). In the third step, according to the comparison results, sequencing data samples were screened that the sequencing depth is more than 10× and the genome coverage is more than 95%. Finally, SNPs of each strain compared with H37Rv were identified using Samtools (https://github.com/samtools/samtools/issues), and the lowest value of comparison quality was set to 30. Then VarScan 2 (http://varscan.sourceforge.net) software was used to further identify and screen SNP fixed mutations with a frequency of more than 75% and supported by at least 10 sequences. The whole genome SNPs detected in this study were compared with known *rpoB* gene mutations (obtained from GenBank gene database) to obtain the mutation information of *rpoB* gene of each strain [12], and only the non-synonymous mutations were recorded.

### MIRU loci repetitive number determination

The sequencing data outputted from Fastp were assessed by FastQC (http://www.bioinformatics.babraham.ac.uk/projects/fastqc) to guarantee good reads quality. Spades (https://github.com/ablab/spades) was carried to assemble second generation sequencing data to long sequence, the finally assembled data were assessed by QUAST (http://bioinf.spbau.ru/quast) and BUSCO (https://busco.ezlab.org). MIRUReader (https://github.com/phglab/MIRUReader) was used to get the repetitive number of 24 MIRU loci (MIRU02, MTUB04, ETRC, MIRU04, MIRU40, MIRU10, MIRU16, MTUB21, MIRU20, QUB11B, ETRA, Mtub29, Mtub30, ETRB, MIRU23, MIRU24, MIRU26, MIRU27, Mtub34, MIRU31, Mtub39, QUB26, QUB4156, MIRU39) directly from long sequence reads [9].

### HGDI calculation

$$HGDI=1-\frac{1}{N\left(N-1\right)}{\sum}_{j=1}^s nj\left(j-1\right)$$

N stands for the total number of strains, nj is the number of strains with the jth genotype, and s is the number of different genotypes at the MIRU-VNTR loci.

### Statistical analysis

IBM SPSS 18.0 and GraphPad 7 were implemented for statistical analysis. Chi-square test or t-test was conducted to compare the differences in variables of general characteristics between TB groups with mutational and non-mutational *rpoB* gene. All variables with a *P*-value < 0.10 on Chi-Square test and t-test were included in a multivariate conditional logistic regression model to investigate the relationship of the mutation of *rpoB* gene and the repetitive number of the MIRU loci. Besides, Cochran-Armitage analysis was conducted to determine the trend of *rpoB* gene mutation rate and the repetitive number of MIRU loci.

## Results

### The rpoB mutation results of the study samples

There were 424 TB samples included in our study, 357 (84.2%) strains extracted from them were RR-TB, and 67 (15.8%) were rifampicin sensitive strains. Among RR-TB, *rpoB* genes of 304 strains were detected mutations, and 6 of 67 susceptible strains were detected mutations. *RpoB* gene mutational rate between strains of retreated cases (83.41%) and new cases (62.32%) showed a significant difference (χ^2^ = 24.0 *P* < 0.05)*.*

### Relation between rpoB gene and 24-loci MIRU-VNTR

**Fig. 1:**
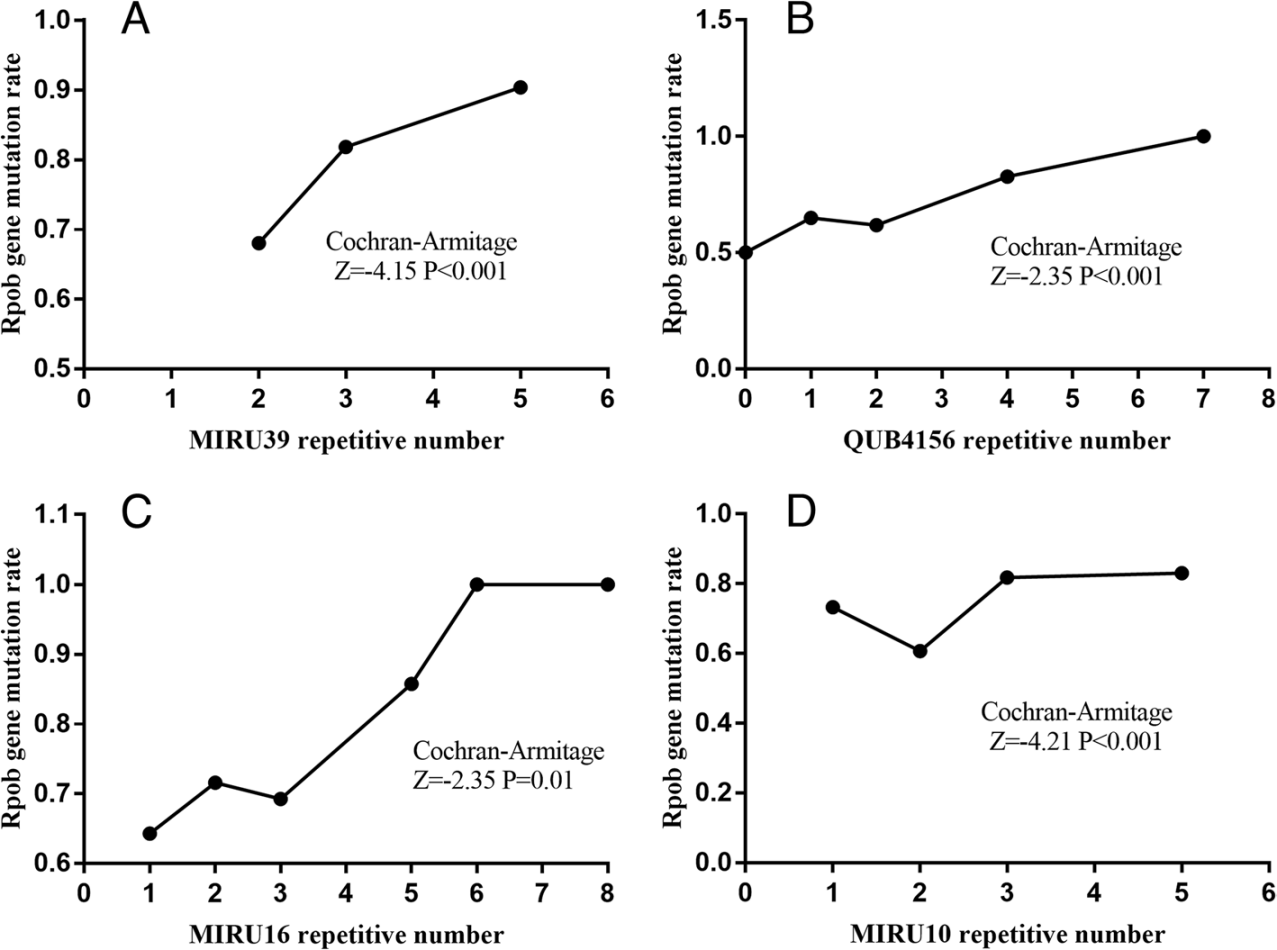
Cochran-Armitage analysis of *rpoB* gene mutation rate and MIRU repeated numbers. The abscissa of the black dot on the broken line represents the repetitive number of different MIRU loci, and the ordinate represents the corresponding *rpoB* gene mutation rate. The broken line trend reflects whether the *rpoB* mutation rate increases or decreases with the increase of MIRU loci repetitive number. When *P* < 0.05, there was a significant overall trend between them

The mutation rate of *rpoB* gene showed an upward trend with the increase of MIRU10, MIRU39, QUB4156 and MIRU16 repetitive number after the Cochran-Armitage analysis (Fig. 1). However, only the repetitive number of MIRU10, MRIU39 and QUB4156 were risk factors for *rpoB* gene mutation after adjusted by category (retreated or new cases) and MIRU23 (Table 1).

**Table 1:** Logistic regression analysis of the *rpoB* gene mutation and the repetitive number of 24 MIRU loci

Variables		Unadjusted			Adjusted*	
	*OR*	*95%CI*	*P*	*OR*	*95%CI*	*P*
MIRU10	1.409	1.197–1.658	<.001	1.332	1.114–1.591	0.002
MIRU16	1.314	1.042–1.658	0.021	1.101	0.846–1.433	0.475
QUB4156	1.403	1.195–1.648	<.001	1.400	1.057–1.856	0.019
MIRU39	1.656	1.284–2.137	<.001	1.257	1.052–1.501	0.012

Note*adjusted for category (new or retreated case), MIRU23

### Allelic diversity of the MIRU loci

As shown in the Table 2, two loci (MIRU10, QUB4156) were highly discriminative (Hunter-Gaston discriminatory index, HGDI> 0.6), two loci (MIRU39, MIRU16) were moderately discriminative (HGDI> 0.3) among all 24 loci studied. The allelic diversity of the 4 loci were different between the *rpoB* gene mutational strains and non-mutational strains. It was worth noting that MIRU39 showed a moderately discriminablility in *rpoB* gene mutational strains, while a low discriminablility in *rpoB* gene non-mutational strains.

**Table 2:** Allelic diversity of four loci in *rpoB* gene mutation and non-mutation isolates

		Alleles		Most frequently repeat		Alleles diversity	
Loci	HGDI	M	N-M	M	N-M	M	N-M
MIRU10	0.65	4	4	5	2	0.65	0.56
MIRU39	0.39	3	3	2	2	0.45	0.20
QUB4156	0.62	5	4	4	2	0.59	0.66
MIRU16	0.43	6	4	2	2	0.45	0.38

Notes: *M rpoB* gene mutational strains, *N-M rpoB* gene non-mutational strains, *HGDI* Hunter-Gaston discriminatory index

## Discussion

In this study, we assessed the associated risk factors for *rpoB* gene mutation in data sourced areas. The *rpoB* gene mutation rate of retreated TB patients (83.41%) was higher than that of new cases (62.32%), it has been proved that the RIF resistance rate of retreated tuberculosis is higher than that of new cases in previous studies [13, 14], the higher rate may since that patients with retreated pulmonary tuberculosis often fail in the initial treatment due to unreasonable or irregular anti-tuberculosis treatment, resulting in the dominant growth of drug-resistant tuberculosis bacteria, and it’s drug resistance mechanism is related to the mutation of *rpoB* gene which coding RNA polymerase β-subunit [3]. Notably, we found no *rpoB* gene mutation in partial RR-TB strains, but mutations in sensitive strains, the inconsistency between gene resistance and phenotype resistance may be caused by heterogeneity of *Mtb.* The presence of low-frequency RR-TB and the predominance of sensitive *Mtb* in the specimen may result in ineffective extraction of drug-resistant DNA if the specimen is not handled properly, while the proportional method of drug sensitivity suggested that it was RR-TB [15]. Patients may have been treated with multiple anti-tuberculosis drugs before sputum specimens were sent for testing, resulting in multiple *Mtb* states in sputum specimens, which can also lead to this result [16]. And mutations in the *rpoB* gene leading to low levels of rifampicin resistance may be the reason that these strains with mutations in the *rpoB* gene were detected as sensitive [17].

Different VNTR loci always has different discrimination ability between Beijing and non-Beijing genotype Mtb [18]. In our study, MIRU10, MIRU39, QUB4156 and MIRU16 all showed a difference in allellic diversity between the Beijing and non-Beijing genotype strains, but only MIRU39 showed remarkable difference (△HGDI > 0.2).

VNTR is a highly polymorphic and highly repetitive DNA fragment, which is characterized by variety and wide distribution. The distribution of VNTR in *Mtb* showed high individual specificity [19]. In recent years, MIRU-VNTR had been widely used in the typing of tuberculosis, some loci, such as MIRU10, MIRU39 and QUB4156 could genotype *Mtb* with high discriminatory power [20–22]. In this study, we found that strains with high MIRU10, MIRU39 QUB4156 or MIRU16 repetitive numbers may often have a high *rpoB* gene mutation rate, but only the repetitive number of MIRU10, MRIU39 and QUB4156 were risk factors for *rpoB* gene mutation after adjusting by category (retreated or new cases) and MIRU23.

MIRU loci are located in the spacer of DNA coding genes, and their specific functions are not clear. Some scholars [23–25] believed that the difference in the copy number of MIRU sites upstream of the coding gene will lead to the difference in the number of ribosomal binding sites (RBS), thus affecting the transcription and expression level of the gene. The coding product encoded by the *fadB* gene downstream of MIRU10 is an oxidoreductase that binds to flavin adenine dinucleotide (FAD), the oxidative stress response induced by this gene may be one of the mechanisms of anti-tuberculosis drugs killing bacteria [26]. With the increase of MIRU10 loci repetitive number, it may increase the inhibition of *fadB* gene expression [27], finally resulting in RIF resistance. *EccCa1* gene which downstream of MIRU39 is part of the ESX-1 specialized secretion system, which delivers several virulence factors to host cells during infection, including the key virulence factors ESAT-6 and CFP-10 [28, 29]. The increase of MIRU39 repetitive number may target up-regulation of *eccCa1* gene expression, resulting in increased bacterial virulence. The coding product encoded by the murT gene downstream of QUB4156 is involved in the pathway peptidoglycan biosynthesis, which is part of cell wall biogenesis [30]. The increase of QUB4156 repetitive number may enhance the virulence of *Mtb* by promoting the synthesis of cell wall.

The mutation rate of *rpoB* gene increased with the addition of MIRU10, MRIU39 and QUB4156 repetitive numbers, we speculated that these MIRU loci caused the RIF resistance in Mtb respectively through different ways. Repetitive numbers of MIRU loci are relatively easy to detect in the laboratory [31]. We hope that this upward trend can deepen the understanding of the function of MIRU10, QUB4156 and MIRU39 loci and the mechanism of RIF resistance. However, this experiment is limited to the research characteristics of molecular epidemiology, which need to be further verified by experimental research.

## Conclusion

The mutation rate of *rpoB* gene increased with the addition of the number of repeats at MIRU10, QUB4156 and MIRU39 loci.

## Data Availability

The sequencing datasets used and/or analyzed during the current study are available from NCBI Sequence Read Archive (SRP134826) and Genome Sequence Archive (CRA000786) (https://ngdc.cncb.ac.cn/search/?dbId=gsa&q=CRA000786).
